# Inappropriate non-vitamin K antagonist oral anticoagulants prescriptions: be cautious with dose reductions

**DOI:** 10.1007/s12471-019-1267-9

**Published:** 2019-04-04

**Authors:** M. S. Jacobs, M. van Hulst, Z. Campmans, R. G. Tieleman

**Affiliations:** 1 Department of Clinical Pharmacy and Toxicology, Martini Hospital, Groningen, The Netherlands; 2 Groningen Research Institute of Pharmacy, Unit of PharmacoTherapy, -Epidemiology & -Economics (PTEE), University of Groningen, Groningen, The Netherlands; 3 Department of Health Sciences, University of Groningen, University Medical Center, Groningen, The Netherlands; 4 Department of Cardiology, Martini Hospital, Groningen, The Netherlands; 5 Department of Cardiology, University Medical Center Groningen, University of Groningen, Groningen, The Netherlands

**Keywords:** Anticoagulants, Atrial fibrillation, Direct thrombin inhibitors, Factor Xa inhibitors, Prescription monitoring

## Abstract

**Background:**

Non-vitamin K antagonist oral anticoagulants (NOACs) are prescribed to patients with atrial fibrillation (AF) to reduce the risk of stroke. Prescribing the correct dose warrants careful consideration of the prevailing dose criteria that differ per NOAC. Electronic systems are useful to intercept prescriptions that are incorrect based on simple ‘primary’ criteria, for example dosing frequency and drug-drug interactions with concomitant medication. However, these systems do not take into account patient characteristics such as age, renal function or weight, which are crucial elements to determine the NOAC dose.

**Methods:**

Our goal was to determine the appropriateness of all prescriptions, as compared with the product labelling approved by the European Medicines Agency, to address common pitfalls in prescribing NOACs. AF patients with a first NOAC prescription between January 2012 and December 2016 were identified from our electronic hospital information system (Martini Hospital, Groningen, the Netherlands).

**Results:**

The study included 3,231 AF patients who had started on an NOAC; 10.7% received an inappropriate dose and the appropriateness of the prescription could not be determined in 14.1%. Underdosing and overdosing occurred in 5.4% and 4.5% of all prescriptions, respectively. A reduced-dose NOAC was a predictor for incorrect prescribing (odds ratio: 2.70, 95% confidence interval: 2.13–3.41). Patient factors were identified that predicted incorrect prescriptions for dabigatran and apixaban.

**Conclusion:**

An incorrect prescription occurred more often in the reduced-dose NOAC group. Clinical parameters such as renal function are often unknown whilst these are essential to determine the right NOAC and dose.

**Electronic supplementary material:**

The online version of this article (10.1007/s12471-019-1267-9) contains supplementary material, which is available to authorized users.

## What’s new?

- Incorrect prescribing of non-vitamin K antagonist oral anticoagulants (NOACs) occurs more often in the reduced-dose NOAC group (apixaban 2.5 mg, dabigatran 110 mg and rivaroxaban 15 mg) compared with the full-dose group.
- The renal function was unknown in 13.9% of the patients whilst this is one of the crucial factors to determine the appropriateness of the prescription.
- Older age (≥80 years) was a predictor for incorrect apixaban prescriptions. Reduced renal function and verapamil use were predictors for incorrect dabigatran prescriptions.

## Introduction

The non-vitamin K antagonist oral anticoagulants (NOACs) are prescribed to patients with atrial fibrillation (AF) to reduce the risk of stroke. NOACs have a direct, predictable therapeutic effect allowing a fixed-dose regimen [1–5]. The correct prescribed dose of the NOACs is determined by several patient characteristics and also concomitant medication use. Prescribing the correct NOAC dose warrants careful consideration of the prevailing dose criteria that differ per NOAC. Several larger studies have been carried out to evaluate prescribing patterns of NOACs, focusing on prescription errors. These studies found a large variety in error rates in prescriptions ranging from 9 to 49%, although most were around 9.7% to 28% [6–13]. Computerised physician order entry is generally supported by a basic level of clinical decision support to assure correct prescribing. Electronic systems are useful to intercept incorrect prescriptions based on simple ‘primary’ criteria, for example the dosing frequency and drug-drug interactions. However, these systems do not take into account patient characteristics such as age, renal function or weight, which determine the appropriate choice of a specific NOAC and the dose. Inadequate dosing can influence the effectiveness and safety of these oral anticoagulants. As a case study, we have explored the prescription policy within our large teaching hospital. Our goal was to determine the appropriateness of all prescriptions, as compared with the product labelling approved by the European Medicines Agency (EMA), to explore common pitfalls in prescribing NOACs.

## Method

This study was a retrospective, cohort study on NOAC prescriptions in AF patients who started anticoagulation therapy in our hospital (Martini Hospital, Groningen, the Netherlands) between 1 January 2012 and 13 December 2016. All data were collected from the electronic hospital information system (HiX, Chipsoft, Amsterdam, the Netherlands). The study included all inpatient and outpatient prescriptions. Patients were included in the study if they had at least one NOAC prescription within the study period and had an AF diagnosis in their electronic medical file confirmed by a cardiologist. The first prescription of the following NOACs was included based on the Anatomical Therapeutic Classification (ATC): B01AF02 (apixaban), B01AE07 (dabigatran), B01AF03 (edoxaban) and B01AF01 (rivaroxaban). The index date was the date that an NOAC was initiated.

### Outcomes and definitions

The eligibility of oral anticoagulation for stroke prevention in AF was determined by a patient’s stroke risk by means of the CHA_2_DS_2_-VASc score; women with a CHA_2_DS_2_-VASc score of ≥2 and men with a CHA_2_DS_2_-VASc score of ≥1 were eligible for an oral anticoagulant [14, 15]. The primary outcomes included the percentage of inappropriate prescribing and the reasons for a prescription being classified as inappropriate. A prescription was reported as inappropriate if the patient had ≥1 inappropriate dosing criteria according to the European Society of Cardiology (ESC) guideline for AF and the summary of product characteristics (SmPCs) as registered with the EMA. The dosing criteria that were used to evaluate the appropriateness of the prescriptions are summarised in the online Supplementary Material, Table S1. Prescriptions that were classified as inappropriate solely based on a missing value or multiple missing values were reported as ‘unknown inappropriateness’. All incorrect prescriptions, overdosed or underdosed, were considered ‘actionable interventions’ where the stroke prevention needed optimisation. The action could be a dose reduction, an increase in the dose or a switch to another NOAC if contraindicated.

### Statistical analysis

IBM SPSS Statistics for Windows (Version 20.0. Armonk, NY: IBM Corp.) was used to perform all data handling, to calculate the CHA_2_DS_2_-VASc score and the bleeding score (HAS-BLED), and to perform all statistical analyses. Descriptive statistics included mean and standard deviation (SD) for continuous variables; numbers and percentages were calculated for categorical variables. Logistic regression was performed to determine if there was a difference in incorrect prescriptions based on full-dose (apixaban 5 mg, dabigatran 150 mg and rivaroxaban 20 mg) versus reduced dose (apixaban 2.5 mg, dabigatran 110 mg and rivaroxaban 15 mg) and to explore predictive factors for incorrect prescriptions per NOAC type. The factors included in the regression analyses were all transformed to a binary variable according to the dose criterion, i. e. for dabigatran: age ≥75 years or <75 years; renal function (estimated glomerular filtration rate) >50 or ≤50 ml/min, verapamil use yes/no. Predictive factors were only included if there were at least 10 observations in both categories. A trend analysis on incorrect prescriptions was performed with a Chi-squared test and linear regression. All analyses were considered statistically significant when *p* < 0.05.

## Results

The study identified 3,291 unique patients who were prescribed an NOAC (apixaban 2.5 mg or 5 mg, dabigatran 75 mg, 110 mg or 150 mg, edoxaban 30 mg or 60 mg and rivaroxaban 2.5 mg, 10 mg, 15 mg or 20 mg) between 1 January 2012 and 13 December 2016. There were no patients with a prescription for edoxaban or rivaroxaban 2.5 mg. Of the identified patients, 3,231 (98.2%) met the predefined inclusion criteria. The main reason for exclusion was an unknown initiation date (*n* = 347, 10.5%) or no confirmed diagnosis of atrial fibrillation (*n* = 260, 7.9%). The patient characteristics for all prescriptions and characteristics per subgroup of NOAC are summarised in Tab. 1.

**Table 1:** Characteristics of the patients with a NOAC prescription

characteristics	all patients (*n* = 3,231)	apixaban (*n* = 914)	dabigatran (*n* = 2,131)	rivaroxaban (*n* = 186)
dose – full dose^a^, *n* (%)	2,112 (65.4)	769 (84.1)	1,184 (55.6)	159 (85.5)
– reduced dose^b^, *n* (%)	1,095 (33.9)	145 (15.9)	930 (43.6)	20 (10.7)
– other dose^c^, *n* (%)	24 (0.7)	–	17 (0.8)	7 (3.8)
sex: male, *n* (%)	1,783 (55.2)	484 (53.0)	1,173 (55.0)	126 (67.7)
age (years), median (range)	72.0 (26.0–101.0)	73.0 (31.0–99.0)	71.0 (26.0–101.0)	69.0 (42.0–95.0)
– ≥75 years, *n* (%)	1,279 (39.6)	337 (36.9)	810 (38.0)	53 (28.5)
weight ≤60 kg, *n* (%)	153 (4.7)	52 (5.7)	96 (4.5)	5 (2.7)
renal function (ml/min)^d^, mean ± SD	72.3 ± 18.4	68.9 ± 20.3	73.8 ± 17.4	72.6 ± 16.0
– 30–50 ml/min, *n* (%)	284 (8.8)	133 (14.6)	138 (6.5)	11 (5.9)
– <30 ml/min, *n* (%)	15 (0.5)	10 (1.1)	4 (0.2)	1 (0.5)
– unknown	448 (13.9)	107 (11.7)	304 (14.3)	37 (19.9)
comorbidity, *n* (%) – CVA/TIA	385 (11.9)	106 (11.6)	265 (12.4)	14 (7.5)
– hypertension	1,939 (60.0)	556 (60.8)	1,283 (60.2)	100 (53.8)
– heart failure	274 (8.5)	92 (10.1)	174 (8.2)	8 (4.3)
– vascular disease^e^	543 (16.8)	174 (19.0)	344 (16.1)	25 (13.4)
– diabetes mellitus	526 (16.3)	163 (17.8)	340 (16.0)	23 (12.4)
CHA_2_DS_2_-VASc score, median (range)	3.0 (0.0–8.0)	3.0 (0.0–8.0)	3.0 (0.0–8.0)	2.0 (0.0–8.0)
HAS-BLED score, median (range)	2.0 (0.0–5.0)	2.0 (0.0–4.0)	2.0 (0.0–5.0)	1.0 (0.0–4.0)

*CHA2DS2-VASc* congestive heart failure of left ventricular dysfunction, hypertension, age ≥75, diabetes, thromboembolism or stroke history, vascular disease, age 65–74 years and sex; *CVA* cerebrovascular accident; *HAS-BLED* hypertension, renal or liver failure, stroke history, bleeding history, labile international normalised ratio, age >65, drugs or alcohol; *kg* kilograms; *ml/min* millilitres/minute; *SD* standard deviation; *TIA* transient ischaemic attack

^a^ 5 mg for apixaban, 150 mg for dabigatran, 20 mg for rivaroxaban

^b^ 2.5 mg for apixaban, 110 mg for dabigatran, 15 mg for rivaroxaban

^c^ 75 mg for dabigatran, 10 mg for rivaroxaban

^d^ None of the patients had a renal function <15 ml/min

^e^ Myocardial infarction and peripheral artery diseases

The majority of the patients were prescribed dabigatran (66.0%) of which 43.6% received the reduced dose. Apixaban was prescribed to 28.3% of the patients of which 15.9% received the reduced dose, rivaroxaban was prescribed to 5.7% of the patients of which 10.7% received the reduced dose. Dabigatran 75 mg was prescribed to 17 patients, 0.8% of all dabigatran prescriptions, and rivaroxaban 10 mg to 7 patients, 3.8% of all rivaroxaban prescriptions. The results of the evaluation of appropriate anticoagulant prescribing are summarised in Tab. 2. The prescriptions of dabigatran 75 mg and rivaroxaban 10 mg are not specified as an NOAC subgroup, as these prescriptions were already inappropriate irrespective of the other dosing criteria.

**Table 2:** Categories of primary non-vitamin K oral antagonist prescriptions classified as (unknown) inappropriate including common mistakes in dosing for all patients and per drug type and dose

	all patients^a^ (*n* = 3,231)	apixaban 5 mg (*n* = 769; 23.8%)	apixaban 2.5 mg (*n* = 145; 4.5%)	dabigatran 150 mg (*n* = 1,184; 36.6%)	dabigatran 110 mg (*n* = 930; 28.8%)	rivaroxaban 20 mg (*n* = 159; 4.9%)	rivaroxaban 15 mg (*n* = 20; 0.6%)
inappropriate prescription	345 (10.7)	20 (2.6)	60 (41.4)	117 (9.9)	108 (11.6)	8 (5.0)	8 (40.0)
– underdosed^b^	174 (5.4)	4 (0.5)	60 (41.4)^c^	6 (0.5)	97 (10.4)	–	7 (35.0)
– overdosed^b^	147 (4.5)	16 (2.1)	–	111 (9.4)	11 (1.2)	8 (5.0)	1 (5.0)
unknown appropriateness	454 (14.1)	113 (14.7)	30 (20.7)	147 (12.4)	129 (13.9)	30 (18.9)	5 (25.0)
missing renal function^d^	448 (13.9)	98 (12.7)	9 (6.2)	166 (14.0)	133 (14.3)	30 (18.9)	5 (25.0)
missing weight^e^	124 (3.8)	96 (12.5)	28 (19.3)	–	–	–	–

All results are presented as numbers plus percentage between brackets

*AF* atrial fibrillation; *CHA2DS2-VASc* congestive heart failure of left ventricular dysfunction, hypertension, age ≥75, diabetes, thromboembolism or stroke history, vascular disease, age 65–74 years and sex; *kg* kilograms

^a^ These prescriptions also include dabigatran 75 mg and rivaroxaban 10 mg which are not registered for AF within Europe

^b^ According to the dosing criteria as described in the Summary of Product Characteristics approved by the European Medicines Agency. These categories include: Wrong dosing regimen was classified as underdosed or overdosed (depending on the NOAC dose), prescriptions in patients with CHA2DS2-VASc score of 1 or less for women and a CHA2DS2-VASc score of 0 for men without a planned electrical cardioversion were classified as overdosed and prescriptions with a contraindication based on renal function

^c^ This number includes apixaban 2.5 mg prescriptions with no dose reduction criteria and prescriptions with only one dose adjustment criterion

^d^ Glomerular filtration rate (ml/min) and serum creatinine (mg/dl) for all prescriptions

^e^ Only counted for apixaban prescriptions for this is the only non-vitamin K oral antagonist with weight as a dose reduction criterion.

**Fig. 1:**
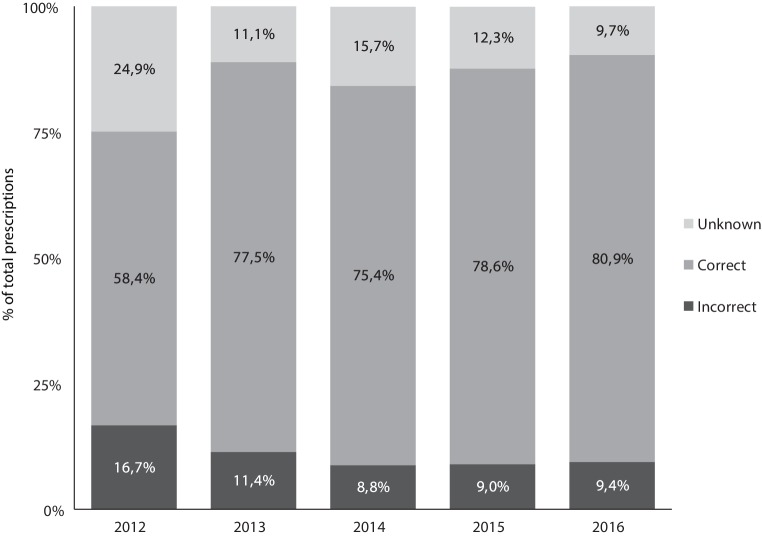
Time trend of correct, incorrect and unknown appropriate prescriptions from 2012–2016

An inappropriate first NOAC prescription was identified in 10.7% of all prescriptions evaluated. All inappropriate prescriptions required action to be taken. Also, 14.1% of all prescriptions were classified as ‘unknown appropriateness’ because crucial information was missing to determine whether the prescription was appropriate or not. The renal function was unknown in 13.9% of all patients and weight (dosing criterion for apixaban) was unknown in 13.6% of all apixaban patients or 3.8% of all patients. Apixaban 2.5 mg was incorrectly prescribed by far the most frequently (41.4%). Apixaban, dabigatran and rivaroxaban were underdosed in 14.8% of the reduced dose prescriptions and overdosed in 6.4% of the full dose prescriptions. A detailed description of the reasons for inappropriate prescribing or unknown appropriateness is listed in the online Supplementary Tables 2–7 per NOAC type and dose. The numbers of correct, incorrect and unknown appropriate prescriptions per year are summarised in Fig. 1. A Chi-squared test showed there was a significant (*p* < 0.001) difference in the number of correct, incorrect and unknown appropriate prescriptions within the study period. Linear regression showed that there was a negative correlation between year prescribed and incorrect prescriptions (*p* < 0.001), indicating that the prescription errors occurred more often at the beginning of the study period in 2012.

**Fig. 2:**
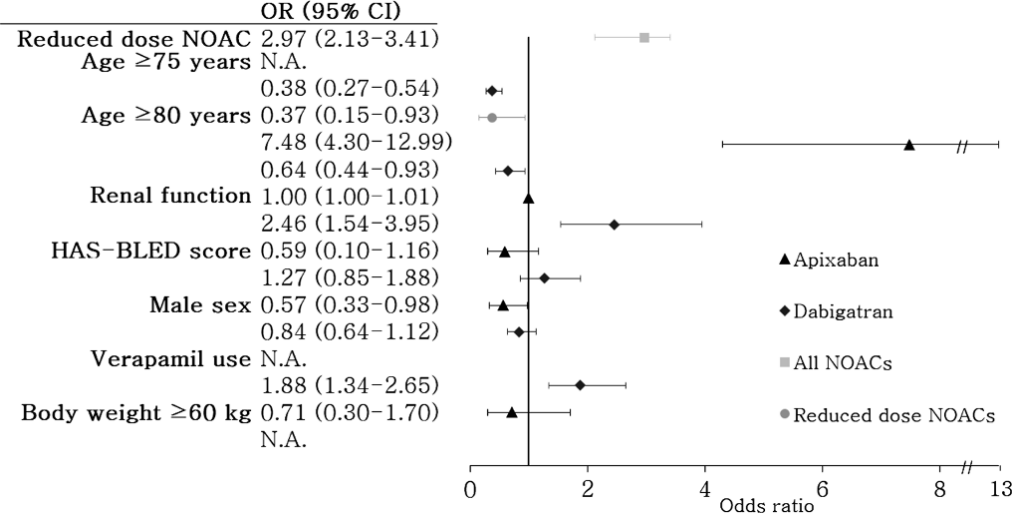
Forest plot of the results of univariate analyses on predictive factors for incorrect prescribing in all NOAC prescriptions and subgroup analyses for apixaban, dabigatran and the reduced dose NOACs

Patients prescribed a reduced dose of the NOAC (apixaban 2.5 mg, dabigatran 110 mg and rivaroxaban 15 mg) had higher odds to receive an incorrect prescription (OR 2.97; 95% CI 2.13–3.41; *p* < 0.01). In the reduced dose NOAC prescriptions, patients aged ≥80 years had lower odds of receiving an inappropriate reduced dose (OR 0.37; 95% CI 0.15–0.93). An analysis of predictive factors was not possible for rivaroxaban due to the small sample size; the analysis for apixaban was limited to variables with enough predictive power to avoid overfitting. In dabigatran prescriptions, an age of ≥75 years had lower odds for an incorrect prescription (OR 0.38; 95% CI 0.27–0.54; *p* < 0.01). The same effect was seen with age ≥80 years as a cut-off (OR 0.64; 95% CI 0.44–0.93; *p* = 0.018). A renal function ≤50 ml/min and verapamil use were predictors for incorrect prescription (OR 2.46; 95% CI 1.54–3.95 [*p* < 0.01] and OR 1.88 95% CI 1.34–2.65 [*p* < 0.01], respectively). The effect of the HAS-BLED score (categorised as high at ≥3), and sex were not significant predictors (*p* = 0.473 and *p* = 0.485, respectively). For apixaban prescriptions, male sex (OR 0.57 95% CI 0.33–0.98; *p* = 0.043) was a predictor for receiving the correct prescription and age ≥80 years (OR 7.48 95% CI 4.30–12.99; *p* < 0.01) was a predictor for receiving an incorrect prescription. A high HAS-BLED score, weight ≥60 kg and serum creatinine ≥133 µmol/l were not significant predictive factors (*p* = 0.124, *p* = 0.712 and *p* = 0.100, respectively). All results are summarised in Fig. 2.

## Discussion

Over the last decade there has been a shift in the prescription of oral anticoagulants, with NOACs now by far the most preferred anticoagulants [16–18]. Evaluating the prescription policy within our hospital over several years revealed important learning points to optimise prescribing but also monitoring of stroke prevention after initiation. Of all 3,231 AF patients who started on an NOAC, 10.7% received an inappropriate dose and the appropriateness of the prescription could not be determined in 14.1%. All incorrect prescriptions were actionable, meaning that the prescription had to be corrected to optimise stroke prevention, with 5.4% of the prescriptions requiring a higher dose and 4.5% of all prescriptions requiring a lower dose. The remaining actionable 0.8% were prescriptions for doses not registered for stroke prevention, dabigatran 75 mg and rivaroxaban 10 mg, and would also require a dose increase. The error rate is fairly low compared with rates found in other studies, possibly because most patients with AF are seen at our specialised AF nurse-led clinic providing integrated chronic care supervised by a cardiologist [19]. Nurse-led structured care of patients with AF has been associated with significantly improved guideline adherence [20]. Another study demonstrated that patient outcomes (cardiovascular-related hospitalisations and death) with nurse-led care were at least as good as in clinical trials [21]. Good outcomes in nurse-led care could very well be due to higher guideline-adherent antithrombotic treatment [22]. The percentage of patients with reduced renal function was relatively low with <10% of the patients having a renal function (eGFR) <50 ml/min. This could indicate that patients with impaired renal function are still typically prescribed a vitamin K antagonist. A reduced dose NOAC was a significant predictor for an incorrect prescription and percentage-wise this was the highest for apixaban 2.5 mg. The same pattern in prescription errors was seen in previous studies [23–26]. In our study, the prescription of a reduced dose was underdosed in 10.4–41.4% of the reduced dose prescriptions. Patients who were prescribed an NOAC in a reduced dose had an OR of 2.97 for receiving an incorrect prescription. This could indicate that prescribers are hesitant to prescribe the full NOAC dose. The use of a reduced dose NOAC without the presence of any dose-reduction criteria could lead to a sub-optimal reduction of the stroke risk, although this has not been studied extensively [13, 27]. Noteworthy, a study in Korean AF patients demonstrated that guideline-discordant dabigatran 110 mg (*n* = 183) had a similar efficacy and safety compared with dabigatran 150 mg (*n* = 294) [28]. The effect of age on receiving an incorrect dose in apixaban and dabigatran prescriptions also illustrates that physicians tend to choose the low dose. The finding that patients aged ≥80 years had lower odds of receiving an inappropriate dose reduction compared with younger patients confirms this hypothesis. This result was mainly driven by the large proportion of dabigatran prescriptions. Age is not a single criterion for apixaban dose reduction and was found to be indicative for an incorrect dose. The high OR for age in incorrect apixaban prescriptions shows that age is often used as a single criterion for dose reduction in apixaban while this is only true in the presence of a low body weight or renal dysfunction (2 out of 3 criteria). Because age is a single dose reduction criterion for dabigatran, a low OR for incorrect prescriptions was found for advanced age. A high bleeding risk, HAS-BLED score ≥3, was not identified as a significant predictor for incorrect apixaban or dabigatran prescription. The ESC explicitly mentions that the bleeding risk should be evaluated; however, a high bleeding risk should not be a reason to withhold oral anticoagulation [14]. With the prescription of NOACs shifting more towards primary care, there will be a transition period in which more attention has to be paid to the prescribing and monitoring. The same tailor-made monitoring tools within a hospital can also be used in primary care.

### Recommendations for improving prescribing

The key to correct prescribing of NOACs in AF patients is the full availability of patient information and the incorporation of all these patient-specific characteristics into the decision making. Ideally, the NOAC can only be prescribed if all necessary information is available. The physician should at least have information on age, weight, comorbidities, renal function (serum creatinine) and concomitant medication. The pharmacist responsible for checking the medication should also have full access to this information, which is required for them to check the prescription before dispensing. Preferably, the patient’s medical history and comorbidities should be registered in the electronic system in such a way that these factors can be computerised into categorical variables and used to monitor the appropriateness of the NOAC dose at a population level. One of the variables most commonly unavailable was renal function, although this is an important factor for determining the correct dose and also for determining a possible contraindication. The renal function can decrease rapidly, especially in older patients, hence periodic monitoring is important to determine if dose adjustment is necessary. Guideline adherence to treatment initiation can still be improved and is a good starting point to decrease the extent of undertreatment and overtreatment [29].

All physicians who prescribe NOACs should be aware of the most common prescription mistakes reported in the present study, namely: 1) inappropriate dose reduction in apixaban patients, especially in those >80 years; 2) inappropriate full-dose dabigatran in patients with using either verapamil or with an impaired renal function. A pharmacist could also assist in the prescription process to further reduce drug-related problems [30]. With the experience of several years of NOAC prescribing, more attention now needs to be paid to achieving and maintaining optimal stroke prevention in the future.

## Conclusion

A hospital-based population-level review of first prescriptions of NOACs showed that in at least 10% of these prescriptions interventions were required to optimise stroke prevention. Prescription errors occurred more often in the reduced dose NOAC group (apixaban 2.5 mg, dabigatran 110 mg and rivaroxaban 15 mg). Clinical parameters such as renal function are often unknown whilst these are essential to determine the right NOAC type and dose.

## Caption Electronic Supplementary Material

Supplementary tablets with a detailed overview of reasons for inappropriate or unknown appropriate prescriptions. The different NOACs and dose have their own table with the dosing criteria according to the drug label.
