# Supplementary material for: Inappropriate non-vitamin K antagonist oral anticoagulants prescriptions: be cautious with dose reductions
Source: Neth Heart J. 2019 Apr 4;27(7-8):371–7. doi: 10.1007/s12471-019-1267-9 (PMC6639841; doi:10.1007/s12471-019-1267-9)
Supplement: Supplementary file 1 — Supplementary tablets with a detailed overview of reasons for inappropriate or unknown appropriate prescriptions. The different NOACs and dose have their own table with the dosing criteria according to the drug label. [file 12471_2019_1267_MOESM1_ESM.docx]

Supplementary tables

Supplementary Table 1: Overview of the full dose, dose-reduction criteria, contraindications and interactions per NOAC

|  | Dabigatran | Rivaroxaban | Apixaban | Edoxaban |
| --- | --- | --- | --- | --- |
| Direct inhibitor of: | Thrombin | Factor Xa | Factor Xa | Factor Xa |
| Full dose | 150 mg bid | 20 mg od | 5 mg bid | 60 mg od |
| Adjusted dose | 110 mg bid | 15 mg od | 2.5 mg bid | 30 mg od |
| Dose-reduction criteria | - ≥80 years - Verapamil - CrCl: 30-50 mL/min | - CrCl: 15-50 ml/min | - CrCl: 15-30 ml/min  Two or more criteria from the following:  ≥80 years, ≤60 kg and/or serum creatinine ≥1.5 mg/dL | - ≤60 kg - CrCl: 15-50 mL/min - P-gp inhibitors* |
| Contraindication | CrCl <30 mL/min | CrCl <15 mL/min | CrCl <15 mL/min | CrCl <15 mL/min |
| Interaction | P-gp inducers and inhibitors | P-gp and CYP3A4 inducers and inhibitors | P-gp and CYP3A4 inducers and inhibitors | P-gp inducers and inhibitors |

Abbreviation: bid = twice daily; CrCl = creatinine clearance; CYP3A4 = cytochrome P450 3A4; NOAC = non-vitamin K antagonist oral anticoagulant; mL/min = milliliters/minute; od = once daily; P-gp = P-glycoprotein.

* cyclosporine, dronedarone, erythromycin and ketoconazole.

Supplementary Table 2: Overview criteria inappropriate prescribing apixaban 2.5 mg

| **Reason for inappropriate prescribing apixaban 2.5 mg (N = 60)** | **N (%)*** |
| --- | --- |
| - No criteria for apixaban 2.5 mg^a^ - Once daily   - Once daily and ≥80 years - Only one apixaban adjusted dose criterion   - Age ≥80 years   - A serum creatinine ≥1.5 milligrams/deciliter   - A weight ≤60 kilograms | **14 (23.3)**  **1 (1.7)**  1  **45 (75.0)**  40  3  2 |
| **Reason for unknown appropriateness apixaban 2.5mg (N = 30)** |  |
| - *Renal function unknown**   - *Only renal function unknown*   - *Renal function unknown and age ≥80 years*   - *Age ≥80 years and weight and renal function unknown* - *At least one apixaban adjusted dose criterion*   - *Age ≥80 years and weight unknown* | **8 (26.7)**  2  5  1  **22 (73.3)**  22 |

* glomerular filtration rate (mL/min) and serum creatinine (mg/dL).

*Unknown appropriateness prescription.*

* Percentage of the number of inappropriate prescriptions or prescriptions with unknown appropriateness

a: A renal function ≥50 mL/min and one or none of the following criteria: age ≥80 years, a body weight ≤60 kg or a serum creatinine ≥1.5 mg/dL for apixaban 2.5 mg.

Supplementary Table 3: Overview criteria inappropriate prescribing apixaban 5 mg

| **Reason for inappropriate prescribing apixaban 5 mg (N = 20)** | **N (%)*** |
| --- | --- |
| Low CHA_2_DS_2_-VASc score^a^  2 criteria for adjusted dose apixaban   - - Age ≥80 years and a weight ≤60 kilograms   - Age ≥80 years and a serum creatinine ≥1.5 mg/dL   - Age ≥ 80 years, a serum creatinine ≥1.5 mg/dL and weight unknown - Wrong dosing regimen - Age ≥80 years, renal function unknown and wrong dose - Age ≥ 80 years, renal function unknown, weight unknown and wrong dose - Wrong dose | **6 (30.0)**  **10 (50.0)**  6  1  3  **4 (20.0)**  1  1  2 |
| **Reasons for unknown appropriateness apixaban 5 mg (N=113)** |  |
| - *Renal function unknown* - At least 1 criterion, 1 or more unknown criterion for adjusted dose apixaban   - Age ≥80 years and weight unknown   - *Serum creatinine ≥1.5 mg/dL and weight unknown*   - *Age ≥80 years and renal function unknown*   - *Weight ≤60 kilograms and renal function unknown*   - *Age ≥80 years and weight and renal function unknown*   - *Weight and renal function unknown* | **65 (57.5)**  **48 (42.5)**  13  4  14  1  3  13 |

Abbreviations: CHA_2_DS_2_-VASc = congestive heart failure of left ventricular dysfunction, hypertension, age ≥75, diabetes, thromboembolism or stroke history, vascular disease, age 65-74 years and sex; mg/dL = milligrams/deciliter; mL/min = milliliters/minute.

* Percentage of the number of inappropriate prescriptions or prescriptions with unknown appropriateness

a: CHA_2_DS_s_-VASc score of 1 or less for women and a CHA_2_DS_2_-VASc score of 0 for men. All patients classified in this category were not planned for electrical cardioversion.

Supplementary Table 4: Overview criteria inappropriate prescribing dabigatran 110 mg.

| **Criteria inappropriate prescribing dabigatran 110 mg (N = 108).** | **N (%)*** |
| --- | --- |
| - Low CHA_2_DS_2_-VASc score^a^ - Only low CHA_2_DS_2_-VASc score - Low CHA_2_DS_2_-VASc score^a^ and renal function unknown - Low CHA_2_DS_2_-VASc score^a^ and no criteria for dabigatran 110 mg^b^ - Wrong dose   - Only wrong dose   - Wrong dose and renal function unknown - No criteria for dabigatran 110 mg^b^ - 1 inappropriate dose criterion:   - Renal function <30 mL/min   2 inappropriate dose criteria:   - Once daily and no criteria for dabigatran 110 mg ^b^ | **11 (10.2)**  6  3  2  **7 (6.5)**  6  1  **88 (81.5)**  **2 (1.9)**  2  **2 (1.9)**  2 |
| **Reason for unknown appropriateness dabigatran 110 mg (N=129)** |  |
| - Renal function unknown   - Only renal function unknown   - Renal function unknown, low CHA_2_DS_2_-VASc score and electrocardioversion scheduled | **129 (100.0)**  126  3 |

Abbreviations: CHA_2_DS_2_-VASc = congestive heart failure of left ventricular dysfunction, hypertension, age ≥75, diabetes, thromboembolism or stroke history, vascular disease, age 65-74 years and sex; mL/min = milliliters/minute.

* Percentage of the number of inappropriate prescriptions or prescriptions with unknown appropriateness

a: CHA_2_DS_s_-VASc score of 1 or less for women and a CHA_2_DS_2_-VASc score of 0 for men.

b: A renal function ≥ 50 mL/min, no concomitant use of verapamil and age <75 years.

**Supplementary Table 5: Overview criteria inappropriate prescribing dabigatran 150 mg.**

| **Criteria inappropriate prescribing dabigatran 150 mg (N = 117).** | **N (%)*** |
| --- | --- |
| - Low CHA_2_DS_2_-VASc score^a^   - Only low CHA_2_DS_2_-VASc score   - Low CHA_2_DS_2_-VASc score^a^ and concomitant use of verapamil   - Low CHA_2_DS_2_-VASc score^a^, and renal function unknown   - Low CHA_2_DS_2_-VASc score^a^ , concomitant use of verapamil, electrocardioversion scheduled - Once daily   - Once daily as only reason   - Once daily and renal function unknown   1 inappropriate dose criterion:   - Concomitant use of verapamil - Concomitant use of verapamil and renal function unknown - Age ≥80 years - Age ≥80 years and renal function unknown - Renal function <50 mL/min   2 inappropriate dose criteria:   - Age ≥80 years and renal function <50 mL/min - Age ≥80 years and concomitant use of verapamil | **34 (29.1)**  24  1  8  1  **6 (5.1)**  4  2  **75 (64.1)**  31  7  18  2  17  **2 (1.7)**  1  1 |
| **Reason for unknown appropriateness dabigatran 150 mg (N=147)** |  |
| - *Renal function unknown*   - Only renal function unknown   - Renal function unknown, low CHA_2_DS_2_-VASc score, electrocardioversion scheduled | **147 (100.0)**  145  2 |

Abbreviations: CHA_2_DS_2_-VASc = congestive heart failure of left ventricular dysfunction, hypertension, age ≥75, diabetes, thromboembolism or stroke history, vascular disease, age 65-74 years and sex; mL/min = milliliters/minute.

* Percentage of the number of inappropriate prescriptions or prescriptions with unknown appropriateness

a: CHA_2_DS_s_-VASc score of 1 or less for women and a CHA_2_DS_2_-VASc score of 0 for men.

Supplementary Table 6: Overview criteria inappropriate prescribing rivaroxaban 15 mg.

| **Criteria inappropriate prescribing rivaroxaban 15 mg (N = 8).** | **N (%)*** |
| --- | --- |
| - No criteria for rivaroxaban 15 mg^a^ - Wrong dose   - Wrong dose and no reason for rivaroxaban 15 mg | **7 (87.5)**  **1 (12.5)**  1 |
| **Reason for unknown appropriateness rivaroxaban 15 mg ( N = 5)** |  |
| - Renal function unknown | **5 (100.0)** |

* Percentage of the number of inappropriate prescriptions or prescriptions with unknown appropriateness

a. Renal function ≥50 ml/min

Supplementary Table 7: Overview criteria inappropriate prescribing rivaroxaban 20 mg.

| **Criteria inappropriate prescribing rivaroxaban 20 mg (N =8).** | **N (%)*** |
| --- | --- |
| - Low CHA_2_DS_2_-VASc score^a^ - 1 inappropriate dose criterion: - Renal function <50 mL/min | **3 (37.5)**  **5 (62.5)**  5 |
| **Reason for unknown appropriateness rivaroxaban 20 mg ( N = 30)** |  |
| - Renal function unknown - Only renal function unknown - Renal function unknown, low CHA_2_DS_2_-VASc score and electrocardioversion scheduled | **30 (100.0)**  28  2 |

Abbreviations: CHA_2_DS_2_-VASc = congestive heart failure of left ventricular dysfunction, hypertension, age ≥75, diabetes, thromboembolism or stroke history, vascular disease, age 65-74 years and sex; mL/min = milliliters/minute.

* Percentage of the number of inappropriate prescriptions or prescriptions with unknown appropriateness

a: CHA_2_DS_s_-VASc score of 1 or less for women and a CHA_2_DS_2_-VASc score of 0 for men
